# 2276: The impact of social influence and impulsivity on IV alcohol self-administration in non-dependent drinkers

**DOI:** 10.1017/cts.2017.125

**Published:** 2018-05-10

**Authors:** Alyssa Schneider, Bethany L. Stangl, Elgin R. Yalin, Jodi M. Gilman, Vijay Ramchandani

**Affiliations:** National Institutes of Health, New York, NY, USA

## Abstract

OBJECTIVES/SPECIFIC AIMS: Impulsivity is a significant predictor of alcohol use and drinking behavior, and has been shown to be a critical trait in those with alcohol use disorder. Suggestibility, or susceptibility to social influence, has been shown to correlate with impulsivity, with highly suggestible individuals being more likely to make impulsive decisions influenced by peer groups. However, the relationship between social influence and drinking behavior is unclear. Our objective was to describe the relationship between social influence and impulsivity traits using the social delayed discounting task and potential differences in intravenous alcohol self-administration (IV-ASA) behavior. METHODS/STUDY POPULATION: Healthy, non-dependent drinkers (n=20) completed a CAIS session, which consisted of an initial 25-minute priming phase, where subjects were prompted to push a button to receive individually standardized IV alcohol infusions, followed by a 125-minute phase during which they could push the button for additional infusions. IV-ASA measures included the peak (PEAK) and average (AVG) BrAC and Number of Button Presses (NBP). Participants completed a social delayed discounting task (SDDT), where participants were presented with the choice of a small, sooner (SS) reward or a large, later (LL) reward. Before starting the task, participants chose peers who selected either the impulsive (SI) or non-impulsive choice (S). Intermittently, the peers’ choice was not shown (X) or different choices (D) were selected. Participants also completed the MISS, the Barratt Impulsiveness Scale (BIS-11), UPPS-P Impulsive Behavior Scale, and the NEO personality inventory. RESULTS/ANTICIPATED RESULTS: Participants with higher suggestibility scores had greater NBP, AVG, and PEAK BrAC in the early phase of the IV-ASA session. Higher scores on the MISS were also correlated with higher impulsivity scores including the NEO Neuroticism (N-factor) measure, BIS-11, and UPPS-P. Results also showed that the MISS score was inversely correlated with the percent of impulsive choices in the SDDT, but that this was independent of peers’ impulsive or nonimpulsive choices. DISCUSSION/SIGNIFICANCE OF IMPACT: These results indicate that non-dependent drinkers that were more susceptible to social influence had heavier drinking patterns, higher IV-ASA, and higher scores on impulsivity measures. In addition, individuals that were more susceptible to social influence made more impulsive choices in general, but those choices were not affected by peer decisions during the task. As such, susceptibility to social influence may be an important determinant of impulsive choices, particularly in relation to alcohol consumption.

